# Cancer Mortality in Workers Exposed to Organochlorine Compounds in the Pulp and Paper Industry: An International Collaborative Study

**DOI:** 10.1289/ehp.8588

**Published:** 2006-03-13

**Authors:** David McLean, Neil Pearce, Hilde Langseth, Paavo Jäppinen, Irena Szadkowska-Stanczyk, Bodil Persson, Pascal Wild, Reiko Kishi, Elsebeth Lynge, Paul Henneberger, Maria Sala, Kay Teschke, Timo Kauppinen, Didier Colin, Manolis Kogevinas, Paolo Boffetta

**Affiliations:** 1 International Agency for Research on Cancer, Lyon, France; 2 Center for Public Health Research, Massey University, Wellington, New Zealand; 3 Cancer Registry of Norway, Institute of Population-Based Cancer Research, Oslo, Norway; 4 Stora Enso Oyj, Imatra, Finland; 5 Nofer Institute of Occupational Medicine, Lodz, Poland; 6 Department of Occupational and Environmental Medicine, University Hospital, Linköping, Sweden; 7 Institut National de Recherche et de Sécurité, National Research and Safety Institute, Department of Epidemiology, Vandoeuvre, France; 8 Department of Public Health, Hokkaido University Graduate School of Medicine, Hokkaido, Japan; 9 University of Copenhagen, Copenhagen, Denmark; 10 National Institute for Occupational Safety and Health, Morgantown, West Virginia, USA; 11 Municipal Institute of Medical Research, Barcelona, Spain; 12 University of British Columbia, Vancouver, Canada; 13 Finnish Institute of Occupational Health, Helsinki, Finland

**Keywords:** epidemiology, mortality, neoplasms, organochlorines, pulp and paper industry

## Abstract

The objective of this study was to evaluate cancer mortality in pulp and paper industry workers exposed to chlorinated organic compounds. We assembled a multinational cohort of workers employed between 1920 and 1996 in 11 countries. Exposure to both volatile and nonvolatile organochlorine compounds was estimated at the department level using an exposure matrix. We conducted a standardized mortality ratio (SMR) analysis based on age and calendar-period–specific national mortality rates and a Poisson regression analysis. The study population consisted of 60,468 workers. Workers exposed to volatile organochlorines experienced a deficit of all-cause [SMR = 0.91; 95% confidence interval (CI), 0.89–0.93] and all-cancer (SMR = 0.93; 95% CI, 0.89–0.97) mortality, with no evidence of increased risks for any cancer of *a priori* interest. There was a weak, but statistically significant, trend of increasing risk of all-cancer mortality with increasing weighted cumulative exposure. A similar deficit in all-cause (SMR = 0.94; 95% CI, 0.91–0.96) and all-cancer (SMR = 0.94; 95% CI, 0.89–1.00) mortality was observed in those exposed to non-volatile organochlorines. No excess risk was observed in cancers of *a priori* interest, although mortality from Hodgkin disease was elevated (SMR = 1.76; 95% CI, 1.02–2.82). In this study we found little evidence that exposure to organochlorines at the levels experienced in the pulp and paper industry is associated with an increased risk of cancer, apart from a weak but significant association between all-cancer mortality and weighted cumulative volatile organochlorine exposure.

Pulp and paper production workers have been exposed to a complex mixture of hazardous substances, including known or suspected carcinogens such as wood dust, various wood extracts and associated bioaerosols, reduced sulfur compounds, talc, formaldehyde, combustion products, epichlorohydrin, acid mists, auramine and other benzidine-based dyes, and a range of chlorinated organic compounds (Kauppinen et al. 1997, 2002). The patterns of exposure in the industry are complicated because of the range of different processes that have been used over time in the various stages of pulp and paper manufacture, which together with the relatively small numbers of workers within specific departments has limited the power of epidemiologic studies of mill-based cohorts. A number of studies, nevertheless, have suggested increased risks of gastrointestinal cancers (Henneberger et al. 1989; Milham and Demers 1984), respiratory system cancers (Milham and Demers 1984; Siemiatycki et al. 1986; Toren et al. 1991), and certain lymphatic and hematopoietic neoplasms (Coggon et al. 1997; Matanoski et al. 1998) in pulp and paper industry workers. Despite the large number of studies conducted, there is still uncertainty about the exact nature and extent of cancer risks associated with work in this industry (Toren 1996).

The International Agency for Research on Cancer (IARC) therefore coordinated an international collaborative cohort study to investigate mortality and cancer incidence in the pulp, paper, paperboard, recycled paper, and paper product industries. This study has combined cohorts from 13 countries, consisting of 98,665 workers (2,110,913 person-years), and included the development of a comprehensive database of exposure measurements for the retrospective assessment of study participants’ exposure (Kauppinen et al. 1997, 2002). The results for mortality and incidence in selected national cohorts (Fassa et al. 1998; Henneberger and Lax 1998; Henneberger et al. 1989; Jäppinen and Pukkala 1991; Jäppinen and Tola 1986; Langseth and Andersen 1999, 2000; McLean et al. 2002; Rix et al. 1997, 1998; Sala-Serra et al. 1996; Szadkowska-Stanczyk et al. 1997; Szadkowska-Stanczyk and Szymczak 2001; Wild et al. 1998), and for separate analyses of exposure to sulfur dioxide (Lee et al. 2002) and asbestos (Carel et al. 2002) in the overall cohort, have been reported.

Workers in this industry experience exposure to chlorinated organic compounds, both volatile chlorinated hydrocarbons such as trichloroethylene, perchloroethylene, dichloromethane, and trichloromethane, and non-volatile organochlorine compounds such as chlorophenols and their salts [pentachlorophenol (PCP)], polychlorinated biphenyls (PCBs), and polychlorinated dibenzodioxins (PCDDs) or polychlorinated dibenzofurans (PCDFs). IARC has classified the volatile organochlorines trichloroethylene and perchloroethylene as probably carcinogenic to humans (group 2A) on the basis of limited evidence in humans of excess risks of cancer of the liver and biliary tract, non-Hodgkin lymphoma (NHL), and esophageal and cervical cancer, and dichloromethane and trichloromethane as possibly carcinogenic to humans (group 2B) based on sufficient evidence for carcinogenicity in animals (Siemiatycki et al. 2004). Of the nonvolatile organochlorine compounds, IARC has classified the 2,3,7,8-substituted tetrachlorodibenzo-*para*-dioxins (TCDDs) as carcinogenic to humans (group 1) on the basis of both mechanistic evidence and limited evidence in humans of excess risk of all cancers combined rather than for any specific site cancer; PCBs as probably carcinogenic to humans (group 2A) because of limited evidence of excess cancers of the liver and biliary tract and of lymphatic and hematopoietic tissues; and PCPs and their sodium salts as possibly carcinogenic to humans (group 2B) based on findings of excess risk of soft tissue sarcoma and NHL (Siemiatycki et al. 2004). The aim of the present study was to investigate the association between cancer mortality and exposure to chlorinated organic compounds in the IARC pulp and paper workers cohort.

## Materials and Methods

Workers employed for at least 1 year in the pulp and paper industry in 13 countries during the period 1920–1996 were included in the overall IARC study, with cohorts from Denmark, Finland, France, Japan, New Zealand, Norway, Poland, Spain, Sweden, Scotland, and the United States included in this analysis of organochlorine exposure. Brazil and South Africa were excluded from this analysis because of inadequacies in the quality of mortality data. Cohort members were identified from company personnel records, with work histories available for the full period of employment in that company.

The exposure assessment procedure used for this study has been described in detail elsewhere (Kauppinen et al. 1997, 2002). Briefly, an international panel of industrial hygiene experts used their professional judgment to estimate exposure over different time periods to 27 main agents at the level of department (but not specific job titles within departments) in each of the mills studied, based on detailed company questionnaires on current and historical raw materials and production processes, and < 31,000 existing (mainly unpublished) occupational exposure measurements. Where sufficient measurement data were available, the expected prevalence and level of exposure to specific agents were quantified, and then depending on the range of estimated exposure levels for each agent, limits for low-, medium-, and high-exposure categories were assigned. For these agents, each worker’s weighted cumulative exposure was then estimated by combining the prevalence, level, and duration of exposure. Where only limited measurement data were available, a qualitative assessment of likely, unlikely, or unknown potential for exposure was made.

Exposure to volatile organochlorine compounds was defined as inhalatory exposure to chlorinated solvents or other specified compounds (indicator agents being trichloroethylene, perchloroethylene, dichloromethane, and trichloromethane) at a level exceeding the nonoccupational background level. These substances have been used as cleaning and degreasing agents in most departments, with the highest exposures occurring in maintenance, repair, and cleaning operations, and medium exposures in sulfate pulp and pulp bleaching departments. The high-exposure category was defined as workers in those departments in which > 50% were exposed and in which the mean level of exposure over the work year was estimated to exceed 1 ppm for trichloromethane, 2.5 ppm for perchloroethylene, and 5 ppm for dichloromethane and trichloroethylene [all 1/10th of the threshold limit value of the American Conference of Governmental Industrial Hygienists (ACGIH 2005)].

Nonvolatile organochlorine exposure was assessed in qualitative terms as ever/never exposed, with exposure defined as potential dermal or inhalatory exposure to PCP or its salts, PCBs, PCDDs, PCDFs, or other non-volatile organochlorine compounds exceeding the nonoccupational long-term background level. PCP has been used to prevent sapstain in softwoods used for pulp, with consequent worker exposure during pulping operations. Exposure to PCBs is most likely to have occurred during maintenance and repair of electrical or hydraulic equipment. Both PCP and PCBs contain PCDDs and PCDFs as contaminants of their manufacture, and PCDDs and PCDFs may also be formed as by-products of the bleaching of pulp with chlorine compounds, resulting in occupational exposure both during bleaching and downstream for those involved in paper production (Krishnan 1990).

Workers were followed up for mortality according to procedures specific to each country. The period of follow-up varied among countries, ranging from 12 to 50 years. Details on periods of employment and follow-up are reported in Table 1. Causes of death were either abstracted from death certificates or obtained from mortality registries, and coded according to the *International Classification of Diseases*, *9th Revision* (ICD-9) [World Health Organization (WHO) 1975]. Tabulation of person-years started at the beginning of the observation period or on day 1 of the second year of employment if this occurred after the start of the observation period. Standardized mortality ratios (SMRs) were calculated as the ratio of observed to expected deaths, with expected deaths being computed by multiplying the person-years in each sex-specific, age-specific, and 5-year calendar-period–specific stratum by the national reference rates using the Person Years program (Coleman et al. 1986). National rates were derived from the WHO Mortality Database (WHO 2001). Ninety-five percent confidence intervals (CIs) of the SMR were calculated under the assumption that the observed numbers of deaths follow a Poisson distribution. Internal analyses were conducted according to years since first employment (< 18, 18–27, 28–37, > 38 years) and duration of exposure (< 4, 4–10, 11–21, > 22 years), and for the volatile organochlorines also by cumulative exposure (∑ level × duration; < 3, 3–9, 10–29, > 30 ppm-years) and weighted cumulative exposure (∑ prevalence × level × duration: < 1, 1–17, > 18 ppm-years), with cut points for continuous measurements of exposure set at quartiles or tertiles depending on numbers available. Tests for linear trend in SMRs were performed using a method described by Breslow and Day (1987).

Poisson regression analysis was used to examine internal dose–response relations associated with exposure to volatile organochlorines and to explore the effect of potential confounding factors. Rate ratios (RRs) and 95% CIs derived from the analysis were adjusted for country, sex, age, calendar period, and employment status (i.e., whether person-years accumulated while workers were employed in the companies included in the study). The reference group for each RR was the first level of each variable.

## Results

The distribution of study participants by exposure status and country is shown in Table 1. At the end of follow-up of the overall cohort, 79% of the workers were alive, 18% had died, 2% were lost to follow-up, and 1% had emigrated. Altogether, 60,468 workers (1,347,782 person-years) were classified according to volatile organochlorine exposure status, with 82% of the person-years classified as ever exposed and 17% (i.e., 9,628 workers or 229,434 person-years) as having high exposure. A total of 58,162 workers (1,259,780 person-years) were classified according to nonvolatile organochlorine exposure status, with 45% (i.e., 24,940 workers or 570,135 person-years) classified as ever exposed. There was significant overlap between the two exposure groups, with maintenance workers in particular often experiencing exposure to both volatile and nonvolatile organochlorines.

Cause-specific mortality for the workers classified according to exposure to volatile organochlorines is shown in Table 2. Among exposed workers there was a deficit of all causes of death (9,350 deaths; SMR = 0.91; 95% CI, 0.89–0.93) and of all malignant neoplasms (2,285 deaths; SMR = 0.93; 95% CI, 0.89–0.97). Of the cancers of *a priori* interest, there were reduced SMRs for cancer of the esophagus (45 deaths; SMR = 0.74; 95% CI, 0.54–0.99), liver (33 deaths; SMR = 0.76; 95% CI, 0.53–1.07), and cervix (17 deaths; SMR = 0.99; 95% CI, 0.58–1.59); for neoplasms of lymphatic or hematopoietic tissues (189 deaths; SMR = 0.94; 95% CI, 0.81–1.08); and for NHL (52 deaths; SMR = 0.86; 95% CI, 0.64–1.13). Statistically significant excess mortality from pleural neoplasms was observed in the exposed group (20 deaths; SMR = 2.00; 95% CI, 1.22–3.09), mostly due to the even larger excess observed in the highly exposed subjects (8 deaths; SMR = 3.67; 95% CI, 1.58–7.23) who were maintenance workers also exposed to asbestos. The elevation observed in those never exposed was virtually identical (4 deaths; SMR = 1.91; 95% CI, 0.52–4.90) to the exposed group, possibly also due to asbestos exposure. Other statistically significant findings included excess mortality from cancer of the penis and other male genital organs (7 deaths; SMR = 2.51; 95% CI, 1.01–5.17) in the exposed group, and cancer of other respiratory organs (4 deaths; SMR = 3.84; 95% CI, 1.05–9.84) in the highly exposed group. The results reported here are for the overall cohort including both men and women. In general, there was little difference in the findings between sexes, although the results among women were based on a relatively small number of deaths.

Cause-specific mortality for the workers classified according to exposure to nonvolatile organochlorines is shown in Table 3. All-cause mortality in the exposed workers was below expected (4,622 deaths; SMR = 0.94; 95% CI, 0.91–0.96), as was mortality from all cancer (1,145 deaths; SMR = 0.94; 95% CI, 0.89–1.00). Mortality from the other neoplasms of *a priori* interest was also below expected, including liver cancer (16 deaths; SMR = 0.69; 95% CI, 0.40–1.13), soft tissue sarcoma (4 deaths; SMR = 0.80; 95% CI, 0.22–2.04), lymphatic and hematopoietic tissue neoplasms in general (97 deaths; SMR = 0.99; 95% CI, 0.81–1.21), and NHL in particular (25 deaths; SMR = 0.86; 95% CI, 0.55–1.26). The only neoplasms showing elevated risks were penis and other cancer of male genital organs (5 deaths; SMR = 3.60; 95% CI, 1.17–8.40) and Hodgkin disease (17 deaths; SMR = 1.76; 95% CI, 1.02–2.82), and in both sites this was more than three times the rate observed in the workers never exposed. It is of interest to note, however, that the 3-fold excesses in risk of cancer of the penis and other male genital organs and of Hodgkin disease in workers not exposed to nonvolatile organochlorines are matched by deficits (of a similar magnitude) in risk of pleural and other respiratory cancers in those not exposed.

No consistent pattern of increasing risk with increasing exposure to either volatile or nonvolatile organochlorines was apparent for any cause of death after stratification by duration of employment or by years since first exposure to both volatile and nonvolatile compounds, or by weighted cumulative exposure to volatile organochlorines (data not shown). The Poisson regression analyses stratified according to weighted cumulative volatile organochlorine exposure (shown in Table 4), and adjusted for sex, age, employment status, calendar year, and country, showed a weak (*p* = 0.002) trend of increasing risk of mortality from all cancer combined with increasing weighted cumulative exposure to volatile organochlorines (< 1 ppm-years: RR = 1; 1–17 ppm-years: RR = 1.12; 95% CI, 1.01–1.24; > 18 ppm-years: RR = 1.19; 95% CI, 1.016–1.34). No other site of *a priori* interest showed a statistically significant trend of increasing risk, although nonsignificant increases were suggested for liver cancer and for cancer of the pleura. The risk estimates, and the positive trend, for cancer of the pleura were essentially unchanged after adjustment for either exposure to or high exposure to asbestos.

## Discussion

In this large multicenter historical cohort study, which examined the risks associated with exposure to both volatile and nonvolatile organochlorines in the pulp and paper industry work environment, we found lower than expected overall mortality and all-cancer mortality rates. Internal comparisons based on duration of exposure and time since first exposure showed no consistent exposure–response trends of risk increasing with either volatile or nonvolatile organochlorines. A weak, but statistically significant, trend of increasing risk of all-cancer mortality with increasing weighted cumulative exposure to volatile organochlorines was observed. This finding is similar to the effect seen in other large cohorts with potential exposure to nonvolatile organochlorines contaminated with TCDD (Kogevinas et al. 1997) but has not previously been reported for volatile organochlorine exposure.

As in most historical cohort studies of industrial workers, we found a deficit in overall mortality and cancer mortality in this study compared with rates expected in the national populations. This is common in occupational cohort mortality studies and has been observed in previous studies of pulp and paper workers (Band et al. 2001; Coggon et al. 1997; Matanoski et al. 1998), due to the healthy worker effect that arises because healthy people are more likely to gain employment and to remain in employment (Checkoway et al. 2004). The healthy worker effect is generally weaker for cancer than for other causes of mortality, as observed in this study.

The assessment of exposure was based on a pulp and paper industry exposure matrix developed by an expert team of industrial hygienists familiar with the pulp and paper industry, although relatively few quantitative data were available on organochlorine exposure (Kauppinen 1997). Estimates of organochlorine exposure were therefore based largely on information available from company questionnaires about processes and raw materials used (e.g., time periods when chlorine bleaching was done or when PCBs were used in mill electrical equipment). Work histories were available only at the department level for most of the mills under study, so individual exposure estimates were based on the level and prevalence of exposure in the department worked in rather than on a more specific job title. The inability to take into account heterogeneity of exposure among workers in a department is likely to have resulted in significant nondifferential mis-classification of exposure, resulting in a tendency to underestimate any true elevation of risk associated with exposure. In addition, because the exposure assessment for the non-volatile organochlorines was qualitative, it was possible only to make internal comparisons based on the likelihood of ever being exposed rather than evaluating trends according to cumulative dose.

As with most historical cohort studies, there is also a lack of information on potential lifestyle confounders such as smoking. However, even for lung cancer, the relatively small differences in smoking status between groups of manual workers are unlikely to account for a relative risk of > 1.5 in studies involving a comparison with national mortality rates (Axelson 1978), and the confounding effect is even weaker for internal dose–response analyses (Siemiatycki et al. 1988). It is therefore unlikely that there is serious confounding by lifestyle factors in the present study, even regarding the findings for pleural cancer and other respiratory cancers. It is possible that the weak but statistically significant exposure–response relationship for all cancers associated with weighted cumulative exposure to volatile compounds could be due to confounding by lifestyle factors or to the exposure of many maintenance workers to other carcinogens, including asbestos.

Overall, we found little evidence of any increased risk of cancer mortality in pulp and paper workers exposed to organochlorines, apart from the weak but statistically significant exposure–response trend for cumulative exposure to volatile organochlorines. Although there is evidence of such an association for all cancers with exposure to phenoxy herbicides contaminated with TCDD (Kogevinas et al. 1997), we are not aware of any evidence suggesting such an association for volatile organochlorine exposure of the type that occurs in pulp and paper mills. Although statistically significant, the association was relatively weak, and this finding should therefore be regarded as preliminary and requiring further investigation.

We found little evidence of increased risks for specific cancer sites that have been previously associated with organochlorine exposure, including cancer of the esophagus, liver, cervix, and NHL for volatile organochlorines, and cancer of the liver, soft tissue sarcoma, lymphatic, and hematopoietic tissue and NHL for nonvolatile organochlorines. Instead, the only sites that showed significant excess risks in those exposed to volatile organochlorines were cancer of the pleura, “other respiratory,” and “penis and other male genital organs,” and for all three sites the risk was higher in those with high exposure. Interestingly, although the excess risk for cancer of penis and other male genital organs was also elevated in those with exposure to non-volatile organochlorines, the risks for cancer of the pleura and other respiratory cancers were elevated only in those never exposed to nonvolatile organochlorines and below expected in those with exposure. For pleural cancer, this may be because many of the group classified as exposed to nonvolatile organochlorines were bleach plant operators, whereas the group with volatile organochlorine exposure were predominantly maintenance workers. So it is possible that these findings could be due to concomitant exposure to asbestos, because the highest exposures to these compounds occurred in maintenance, repair, and cleaning operations, and an excess risk of pleural cancer has already been reported in workers exposed to asbestos in this industry (Carel et al. 2002). Unfortunately, joint analyses of asbestos and organochlorine exposure were equivocal because most of the pleural and other respiratory cancers occurred in workers exposed to both factors, and there were insufficient numbers to determine whether there were increased risks in workers exposed to nonvolatile organochlorines alone. When the organochlorine findings were adjusted for asbestos exposure (data not shown), the RRs were essentially unchanged. Although the elevated risk observed for both cancers of penis and other male genital organs and other respiratory cancers was statistically significant, in both cases the CIs were wide and the small number of cases precluded further analysis. It is possible, therefore, that these are chance findings. Increased risks of Hodgkin disease, however, have been consistently reported among pulp mill workers (Milham and Demers 1984; Toren et al. 1996), woodworkers (McCunney 1999), and those with exposure to the herbicides 2,4-D (2,4-dichlorophenoxyacetic acid), 2,4,5-T (2,4,5-trichlorophenoxyacetic acid) and its contaminant TCDD, cacodylic acid, and picloram (Dich et al. 1997; Institute of Medicine 2000). The statistically significant increase in risk observed among workers with exposure to nonvolatile organochlorines in this cohort appears to be consistent with these earlier findings. Because the survival for NHL is relatively good, an analysis of incidence would have been more informative, but unfortunately cancer incidence data were available only from Denmark, Finland, New Zealand, Norway, and Sweden. The lack of incidence data and the small number of cases precluded any further analysis of this association.

In summary, there is little evidence that exposure to organochlorines at the levels experienced in the pulp and paper industry causes an increased risk of cancer, apart from a weakly statistically significant association with weighted cumulative volatile organochlorine exposure. There was little evidence of an increased risk for any specific cancer sites, apart from a statistically significant association between nonvolatile organochlorine exposure and Hodgkin disease and cancer of the pleura. The finding for pleural cancer is consistent with evidence that maintenance workers were exposed to asbestos.

## Figures and Tables

**Table 1:** Distribution of study participants by exposure status and country.

			Volatile organochlorines						Nonvolatile organochlorines			
			Never exposed^a^		Ever exposed^a^		High exposure^b^		Never exposed^a^		Ever exposed^a^	
Country	Employment period	Follow-up period	No.	Person-years	No.	Person-years	No.	Person-years	No.	Person-years	No.	Person-years
Denmark	1920–1992	1943–1993	1,512	27,258	5,762	142,519	2,538	64,274	2,863	61,791	4,813	115,777
Finland	1935–1995	1945–1995	927	28,048	6,964	218,387	653	13,664	4,022	124,524	3,180	97,437
France	1921–1992	1968–1992	386	6,009	3,260	60,402	36	764	2,265	37,902	2,119	40,811
Japan	1946–1996	1976–1996	567	9,880	1,602	29,520	129	2,609	1,097	19,843	1,221	22,396
New Zealand	1955–1992	1980–1992	3,396	34,792	2,583	27,702	1,155	12,388	4,635	47,717	1,067	11,973
Norway	1920–1993	1953–1993	2,386	55,430	16,002	420,322	4,491	120,494	9,942	252,707	6,063	150,908
Poland	1967–1990	1968–1990	3,538	50,850	2,399	37,676	159	2,902	4,252	61,350	1,833	29,421
Scotland	1920–1990	1955–1994	525	12,480	3,534	85,965	26	810	414	7,075	604	11,171
Spain	1955–1991	1970–1992	228	3,946	1,199	21,219	8	80	2,379	48,815	1,729	33,830
Sweden	1920–1990	1955–1991	969	17,329	2,475	53,210	379	8,323	728	16,947	2,151	53,250
United States	1920–1982	1961–1991	147	2,824	107	2,014	54	1,127	625	10,973	160	3,162
Total			14,581	248,846	45,887	1,098,936	9,628	229,434	33,222	689,645	24,940	570,135

a The total number of workers that could be classified by exposure to volatile (60,468) and nonvolatile (58,162) organochlorines differed.

b High exposure is a subset of ever exposure.

**Table 2:** SMRs for selected causes by exposure to volatile organochlorine compounds.

	Never exposed			Ever exposed			High exposure a		
Cause of death (ICD-9 codes)	Obs	SMR	95% CI	Obs	SMR	95% CI	Obs	SMR	95% CI
All causes	2,175	0.88	0.84–0.91	9,350	0.91	0.89–0.93	1,902	0.90	0.86–0.95
All neoplasms (140–208)	524	0.91	0.83–0.99	2,285	0.93	0.89–0.97	517	1.01	0.93–1.10
Oral cavity and pharynx (140–149)	9	0.68	0.31–1.29	33	0.61	0.42–0.86	11	1.09	0.54–1.95
Esophagus (150)	15	0.99	0.56–1.64	45	0.74	0.54–0.99	6	0.57	0.21–1.24
Stomach (151)	60	0.98	0.75–1.26	201	0.83	0.72–0.95	43	0.95	0.69–1.28
Colon (153)	38	0.98	0.69–1.34	140	0.85	0.72–1.01	35	0.90	0.63–1.25
Rectum (154)	18	0.68	0.40–1.07	98	0.91	0.74–1.10	30	1.19	0.80–1.70
Liver (155)	10	0.76	0.37–1.41	33	0.76	0.53–1.07	6	0.83	0.31–1.82
Gallbladder (156)	6	1.24	0.45–2.69	14	0.66	0.36–1.10	4	1.05	0.29–2.70
Pancreas (157)	32	1.07	0.74–1.52	115	0.90	0.75–1.09	19	0.71	0.42–1.10
Larynx (161)	9	1.18	0.54–2.24	29	1.00	0.67–1.43	3	0.65	0.13–1.89
Lung (162)	122	0.85	0.70–1.01	613	1.04	0.96–1.13	125	1.06	0.88–1.26
Pleura (163)	4	1.91	0.52–4.90	20	2.00	1.22–3.09	8	3.67	1.58–7.23
Other respiratory (164–165)	0	0.00	0.00–2.74	10	1.72	0.82–3.15	4	3.84	1.05–9.84
Soft tissue (171)	2	0.88	0.11–3.20	12	1.13	0.59–1.98	4	1.75	0.48–4.48
Melanoma (172)	9	1.02	0.47–1.94	42	1.12	0.81–1.51	6	0.63	0.23–1.37
Breast (174–175)	4	0.94	0.26–2.41	63	0.96	0.73–1.22	6	0.61	0.22–1.32
Cervix uteri (180)	1	0.89	0.02–4.98	17	0.99	0.58–1.59	2	0.67	0.08–2.40
Prostate (185)	44	0.79	0.58–1.07	177	0.86	0.74–1.00	49	1.02	0.76–1.35
Penis and other male genital organs (187)	1	1.46	0.04–8.11	7	2.51	1.01–5.17	2	3.11	0.38–11.2
Bladder (188)	17	0.88	0.51–1.40	87	1.09	0.88–1.35	20	1.07	0.65–1.65
Kidney (189)	12	0.77	0.40–1.34	54	0.77	0.58–1.01	15	0.97	0.54–1.59
Brain (191–192)	16	1.02	0.58–1.66	68	0.98	0.76–1.25	20	1.25	0.76–1.93
Lymphatic and hematopoietic (200–208)	45	0.96	0.70–1.28	189	0.94	0.81–1.08	41	0.92	0.66–1.25
NHL (200, 202)	15	1.12	0.63–1.86	52	0.86	0.64–1.13	11	0.84	0.42–1.51
Hodgkin disease (201)	4	0.94	0.26–2.42	21	1.07	0.66–1.63	4	0.93	0.25–2.39
Multiple myeloma (203)	7	0.76	0.31–1.58	41	1.02	0.73–1.38	9	1.00	0.46–1.90
Leukemia (204–208)	19	0.99	0.60–1.55	75	0.93	0.73–1.17	17	0.95	0.55–1.51
Circulatory system diseases (390–459)	1,042	0.91	0.86–0.97	4,444	0.95	0.92–0.98	877	0.92	0.86–0.98
Respiratory system diseases (460–519)	130	0.77	0.64–0.91	561	0.82	0.75–0.89	97	0.72	0.59–0.88
Digestive system diseases (520–579)	60	0.71	0.54–0.91	299	0.85	0.75–0.95	47	0.68	0.50–0.91
Liver cirrhosis (571)	23	0.77	0.49–1.16	119	0.95	0.79–1.14	24	1.00	0.64–1.48

Obs, observed.

a High exposure is a subset of ever exposure.

**Table 3:** SMRs for selected causes by exposure to nonvolatile organochlorine compounds.

	Never exposed			Ever exposed		
Cause of death (ICD-9 codes)	Obs	SMR	95% CI	Obs	SMR	95% CI
All causes	5,771	0.89	0.87–0.91	4,622	0.94	0.91–0.96
All neoplasms (140–208)	1,434	0.94	0.89–0.99	1,145	0.94	0.89–1.00
Oral cavity and pharynx (140–149)	33	0.92	0.63–1.29	15	0.51	0.29–0.85
Esophagus (150)	27	0.71	0.41–1.03	26	0.78	0.51–1.15
Stomach (151)	146	0.93	0.79–1.10	98	0.89	0.72–1.08
Colon (153)	106	1.04	0.85–1.25	62	0.74	0.57–0.95
Rectum (154)	60	0.87	0.66–1.12	51	0.96	0.71–1.26
Liver (155)	27	0.87	0.57–1.27	16	0.69	0.40–1.13
Pancreas (157)	67	0.84	0.65–1.06	69	1.12	0.87–1.42
Larynx (161)	18	0.92	0.54–1.45	20	1.23	0.75–1.90
Lung (162)	356	0.98	0.88–1.08	314	1.04	0.93–1.17
Pleura (163)	17	2.78	1.62–4.45	4	0.78	0.21–2.01
Other respiratory (164–165)	8	2.11	0.91–4.16	2	0.66	0.08–2.39
Soft tissue (171)	8	1.22	0.53–2.41	4	0.80	0.22–2.04
Melanoma (172)	20	0.82	0.50–1.27	21	1.17	0.72–1.78
Breast (174–175)	21	0.90	0.55–1.37	32	0.89	0.61–1.25
Prostate (185)	117	0.85	0.70–1.02	84	0.93	0.74–1.15
Penis and other male genital organs (187)	2	1.12	0.14–4.05	5	3.60	1.17–8.40
Bladder (188)	50	1.00	0.74–1.32	43	1.09	0.79–1.46
Kidney (189)	41	0.94	0.67–1.27	18	0.53	0.31–0.83
Brain (191–192)	44	1.02	0.74–1.37	28	0.80	0.53–1.15
Lymphatic and hematopoietic (200–208)	112	0.88	0.72–1.05	97	0.99	0.81–1.21
NHL (200, 202)	35	0.93	0.65–1.30	25	0.86	0.55–1.26
Hodgkin disease (201)	7	0.58	0.23–1.19	17	1.76	1.02–2.82
Multiple myeloma (203)	21	0.83	0.51–1.27	20	1.07	0.66–1.66
Leukemia (204–208)	49	0.95	0.70–1.26	35	0.89	0.62–1.24
Circulatory system diseases (390–459)	2,727	0.92	0.89–0.96	2,157	0.99	0.95–1.04
Respiratory system diseases (460–519)	327	0.78	0.69–0.86	266	0.82	0.72–0.92
Digestive system diseases (520–579)	167	0.74	0.63–0.86	166	0.91	0.77–1.05

Obs, observed.

**Table 4:** Mortality from selected causes by weighted cumulative exposure to volatile organochlorines.

	Weighted cumulative exposure to volatile organochlorines^a^								
	< 1 ppm-years^b^		1–17 ppm-years			> 18 ppm-years			
Cause of death (ICD-9 codes)	Obs	RR^c^	Obs	RR	95% CI	Obs	RR	95% CI	Trend^d^
All neoplasms (140–208)	1,006	1	778	1.12	(1.01–1.24)	489	1.19	(1.06–1.34)	0.002
Esophagus (150)	26	1	14	0.94	(0.46–1.90)	5	0.54	(0.19–1.51)	0.289
Liver (155)	18	1	9	1.05	(0.42–2.65)	6	1.45	(0.51–4.16)	0.525
Lung (162)	253	1	236	1.15	(0.95–1.39)	120	1.07	(0.85–1.36)	0.394
Pleura (163)	6	1	6	1.16	(0.36–3.78)	8	2.49	(0.83–7.53)	0.114
Lymphatic and hematopoietic (200–208)	90	1	66	1.09	(0.78–1.54)	33	0.93	(0.61–1.42)	0.859
NHL (200, 202)	25	1	18	1.15	(0.60–2.22)	9	0.97	(0.43–2.20)	0.955

Obs, observed.

a Weighted cumulative exposure (∑ prevalence × level × duration).

b Reference category.

c RR adjusted for sex, age, employment status, calendar year, and country.

d *p*-Value of test for linear trend.
